# Supplementary figures and images for: Plasmid streamlining drives the extinction of antibiotic resistance plasmids under selection for horizontal transmission
Source: PLoS Biol. 2025 Dec 11;23(12):e3003564. doi: 10.1371/journal.pbio.3003564 (PMC12711027; doi:10.1371/journal.pbio.3003564)

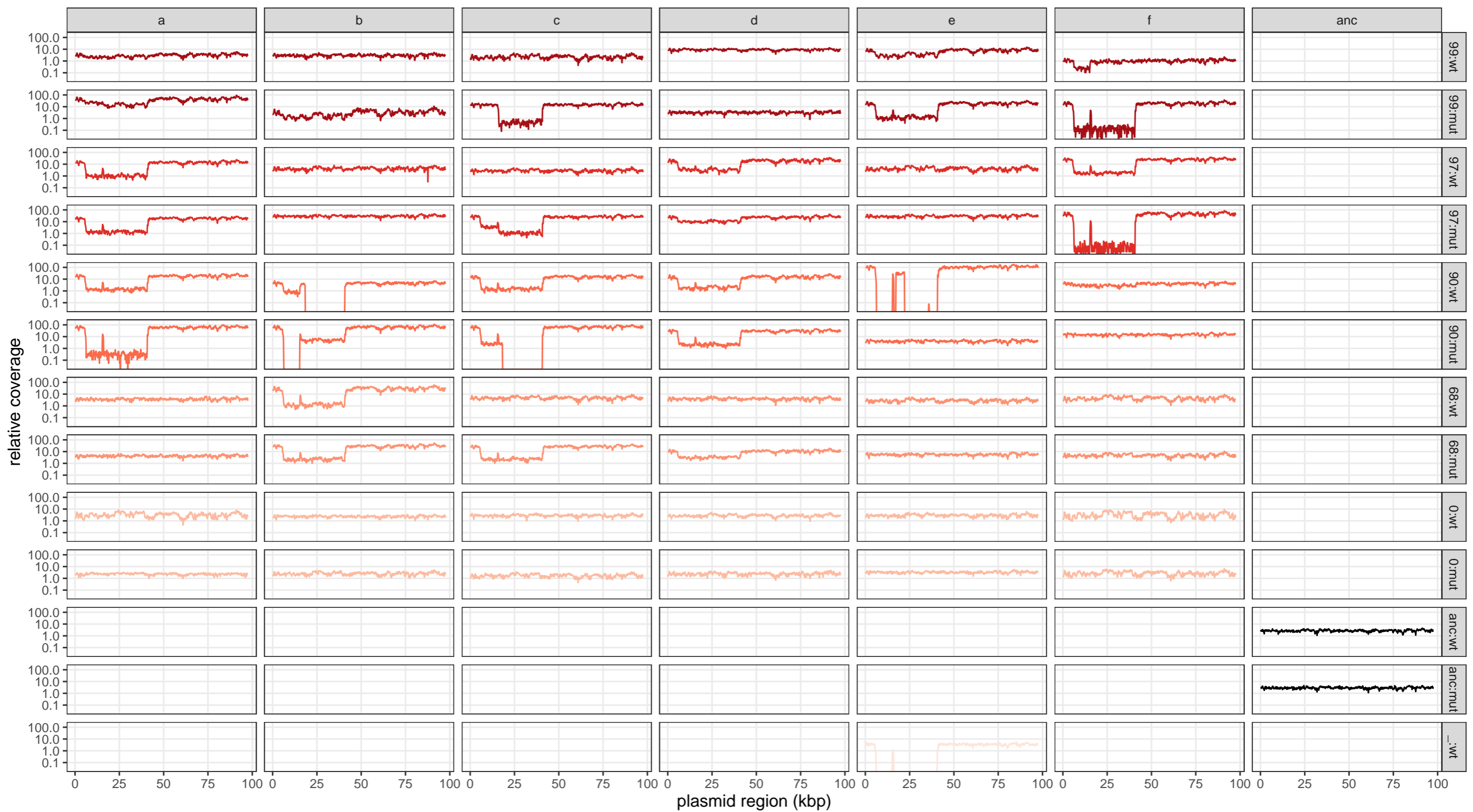

Supplement: S1 Fig — Relative coverage of sequencing reads is shown for all clones across R1wt sequence map. Relative coverage was measured as the sum of coverage of both unique and repeat reads, divided by the overall average coverage of reads mapped to the chromosome. The data underlying this figure can be found in S1 Data. (PDF) [file pbio.3003564.s004.pdf]

**A**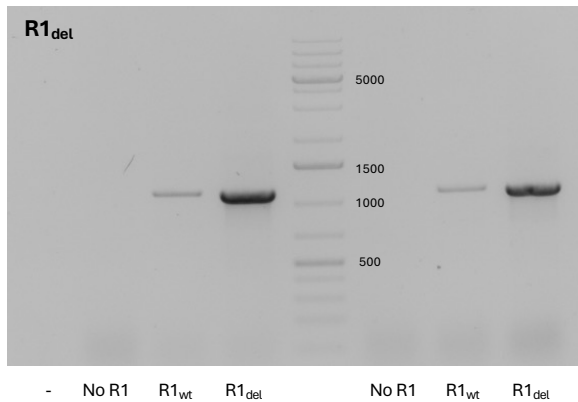**B**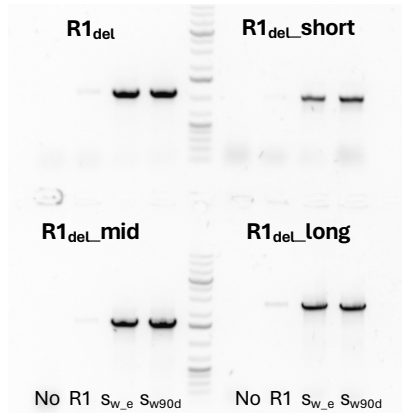**C**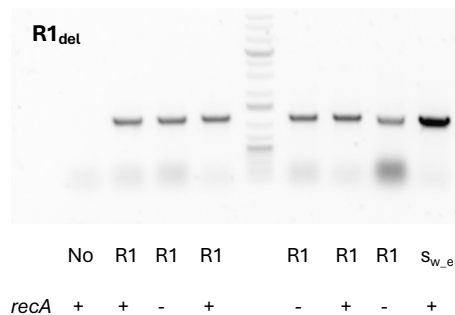**D**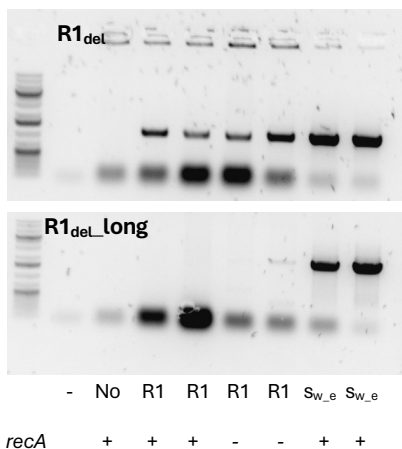

Supplement: S2 Fig — “-” indicates the negative control without bacteria; other reactions were run with Escherichia coli carrying no R1 or R1 variants as indicated. Numbers in A indicate the size of some DNA ladder bands, in bp, the ladder used was the same for all gels. Titles in italics indicate primer pairs used in each section. In A and D, annealing temperature was 64°C; in B and C annealing temperature was 56°C. In A and C, extension time was 15 s; in B and D it was 1 min to ensure full amplification using R1_del_long primer pair. In A to C, 30 cycles were performed; in D 35 cycles were performed to enhance detection of any possible faint band. (PDF) [file pbio.3003564.s005.pdf]

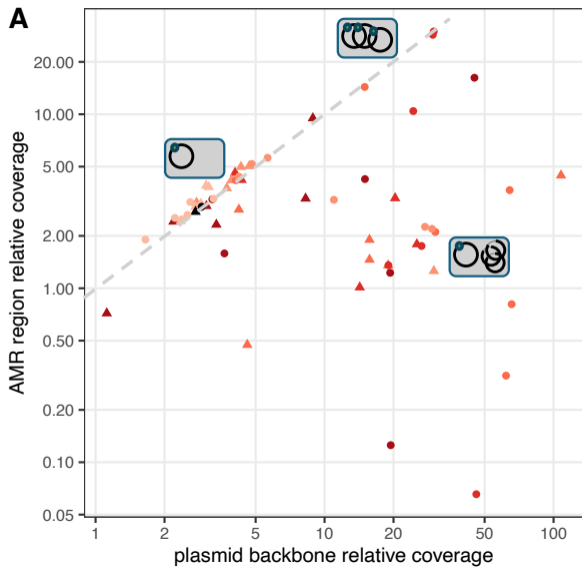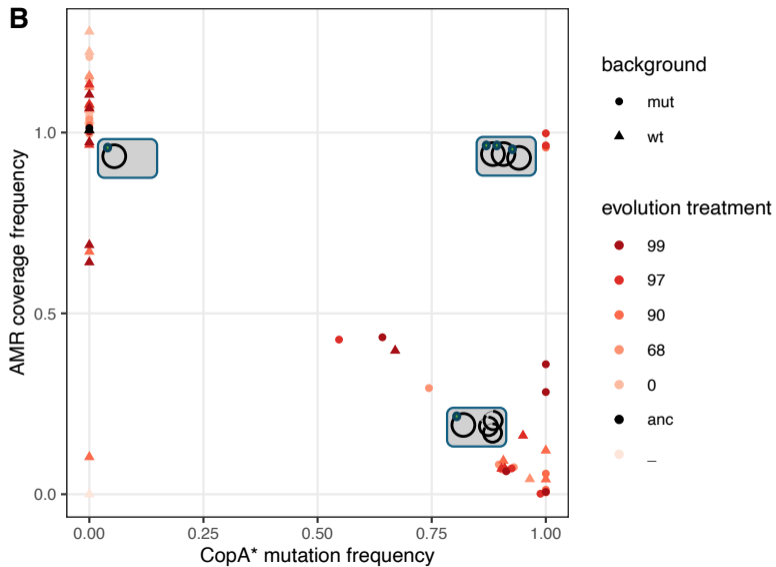

Supplement: S3 Fig — In A, short-read coverage of the AMR region is shown on the y-axis and coverage of the rest of the plasmid is shown on the x-axis; each dot represents a sequenced clone with evolution treatment indicated by color and strain background by dot type (circles = wt, triangles = mut). B shows the relative coverage of the AMR region (compared to the backbone region), as a function of the frequency of copA* mutant allele among sequenced reads for each sequenced clone. Interpretation of plasmid content is shown for three regions of both graphs, with bold lines showing plasmid regions present, thin lines deleted regions, and the green circle indicating the ampicillin resistance marker. The data underlying this figure can be found in S1 Data. (PDF) [file pbio.3003564.s006.pdf]

rep a

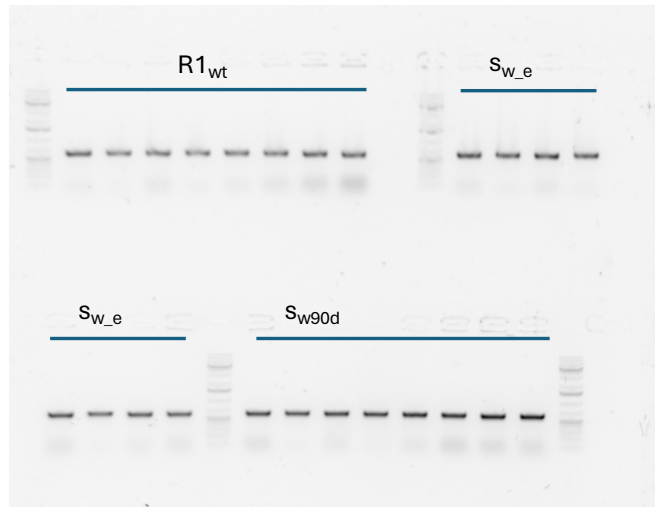

rep b

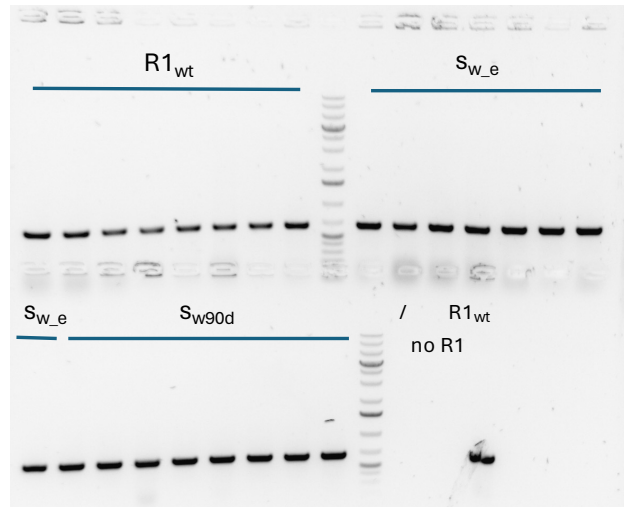

rep c

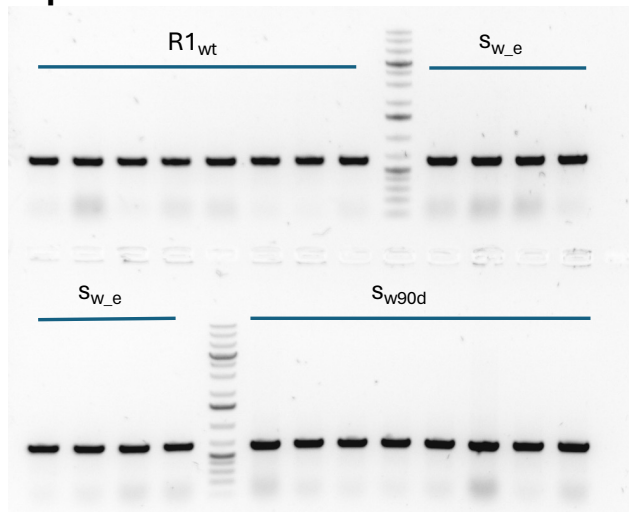

rep d

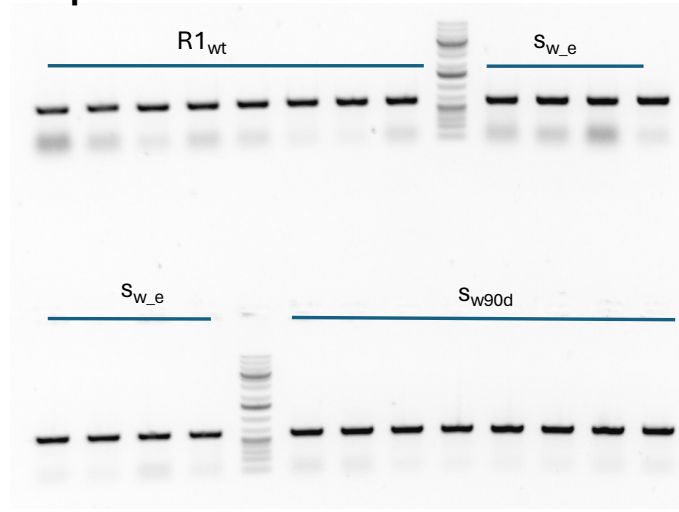

Supplement: S4 Fig — Plasmid presence was checked by colony PCR using parM primers on 8 colonies per evolved population. Each gel shows a population replicate number, with plasmids R1, sw_e and sw90d; rep b gel also includes a no DNA control, a plasmid-free bacteria control and a R1-carrying control, as indicated. (PDF) [file pbio.3003564.s007.pdf]

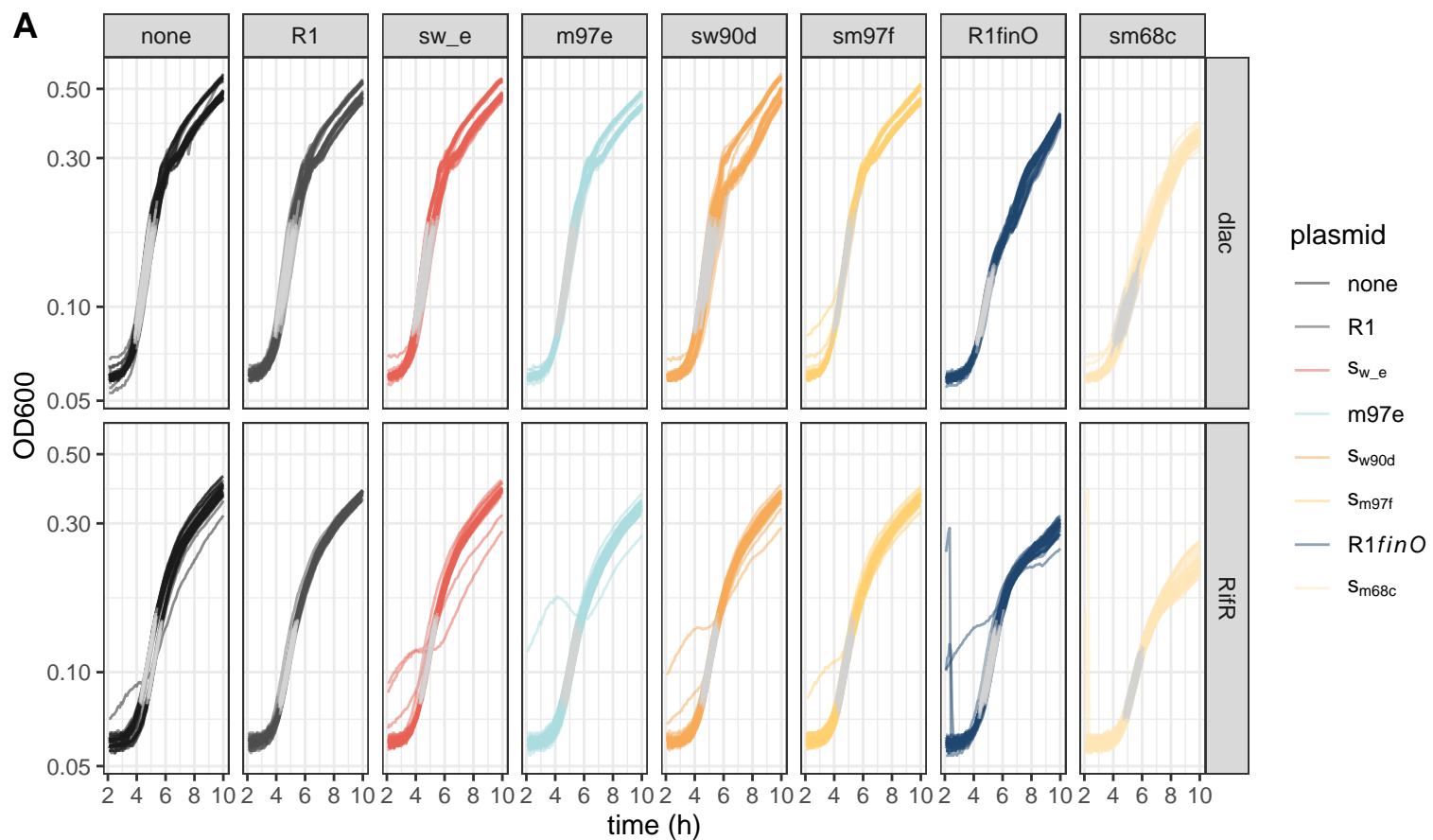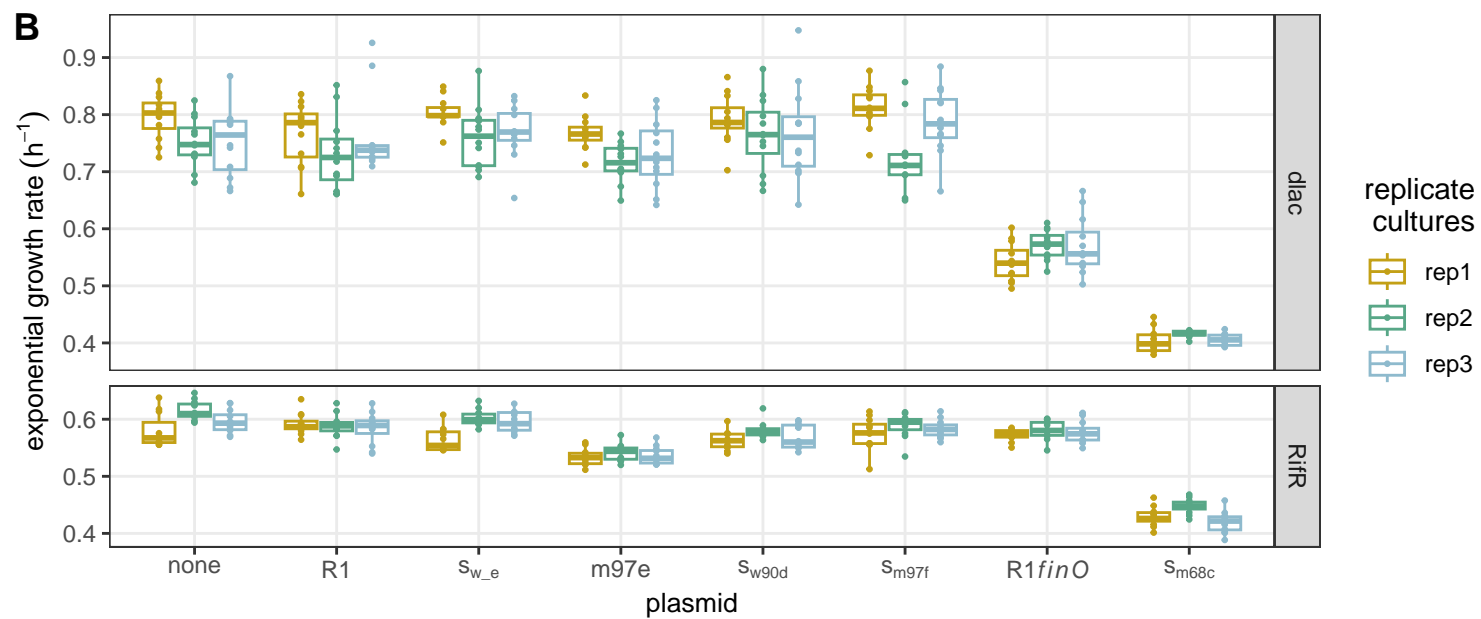

Supplement: S5 Fig — In A, growth curve data are shown for each plasmid in color. The portion of the curves used for calculating exponential growth rate is shown in light gray. In B, calculated exponential growth rates are shown for all strains. Each data point corresponds to an individual technical replicate (well within a 96-well plate); the center line of the boxplots shows the median, boxes show the first and third quartile, and whiskers represent 1.5 times the interquartile range, calculated across N = 12 technical replicates, with different colors indicating independent biological replicates using different overnight cultures. The data underlying this figure can be found in S1 Data. (PDF) [file pbio.3003564.s008.pdf]

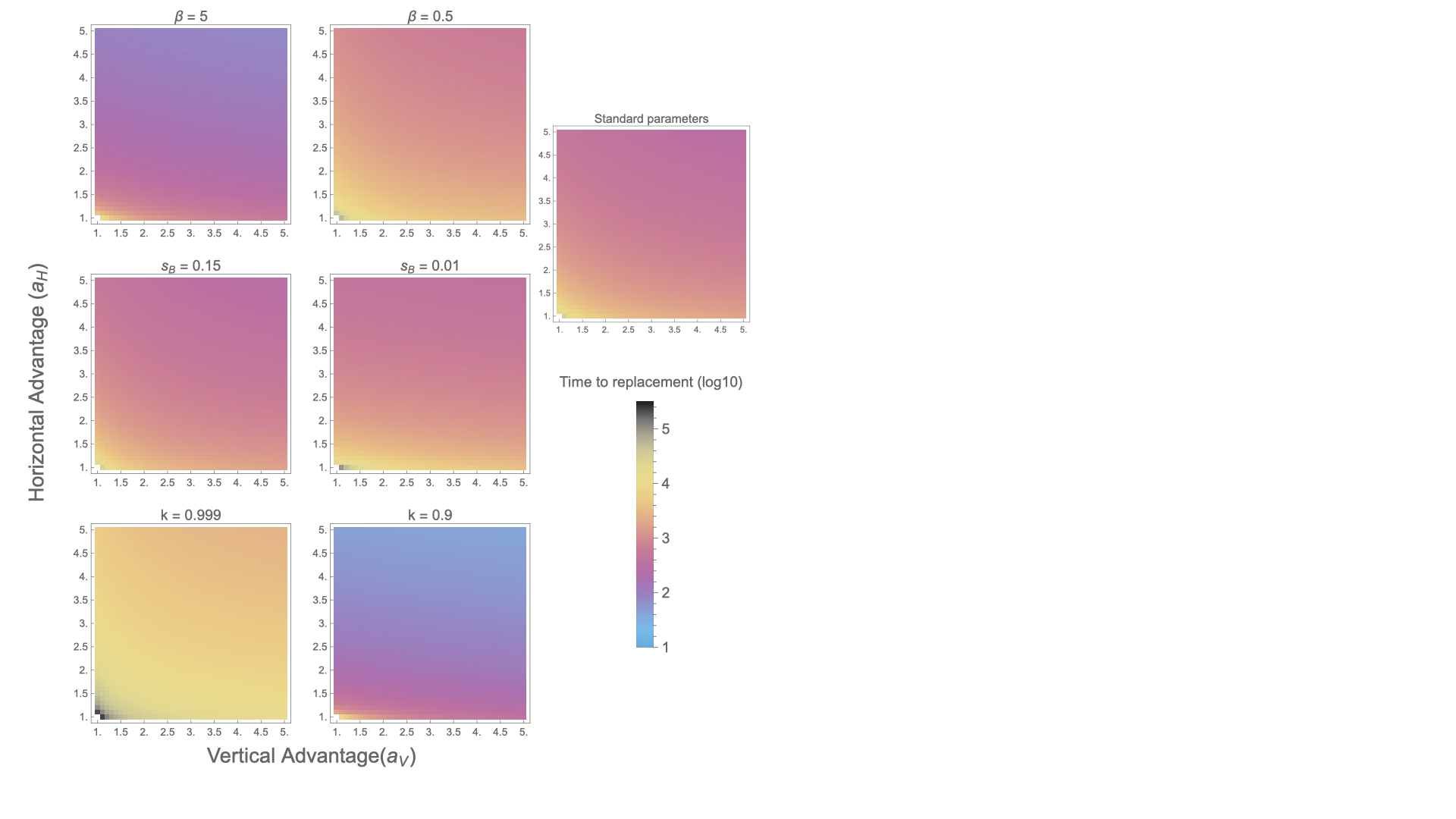

Supplement: S6 Fig — The heatmaps shows the time (in arbitrary units, log10 transformed) taken for the mutant plasmid to replace the wildtype plasmid (defined as the frequency of the wildtype falling below 1%) as a function of horizontal and vertical advantage. Each row shows the impact of setting one parameter differently from the standard values used in the main text. Top: transmission rate of the wildtype plasmid (βW). Middle: partitioning loss of the mutant plasmid (sM). Bottom: Entry exclusion (k). We also show the log10 transformed main text results (standard parameters) for reference. Standard parameter: ρ=1, c=110,γ=110,K=1,βW=1, sM=0.1, k = 0.99, with βM=aHβW and sW=aVsM. We are interested in qualitative insights; the parameters are in arbitrary time units. White indicates no replacement. We have used a log scale to fully capture the extent of variation. The data underlying this figure can be found in S2 Data. (TIFF) [file pbio.3003564.s009.tiff]

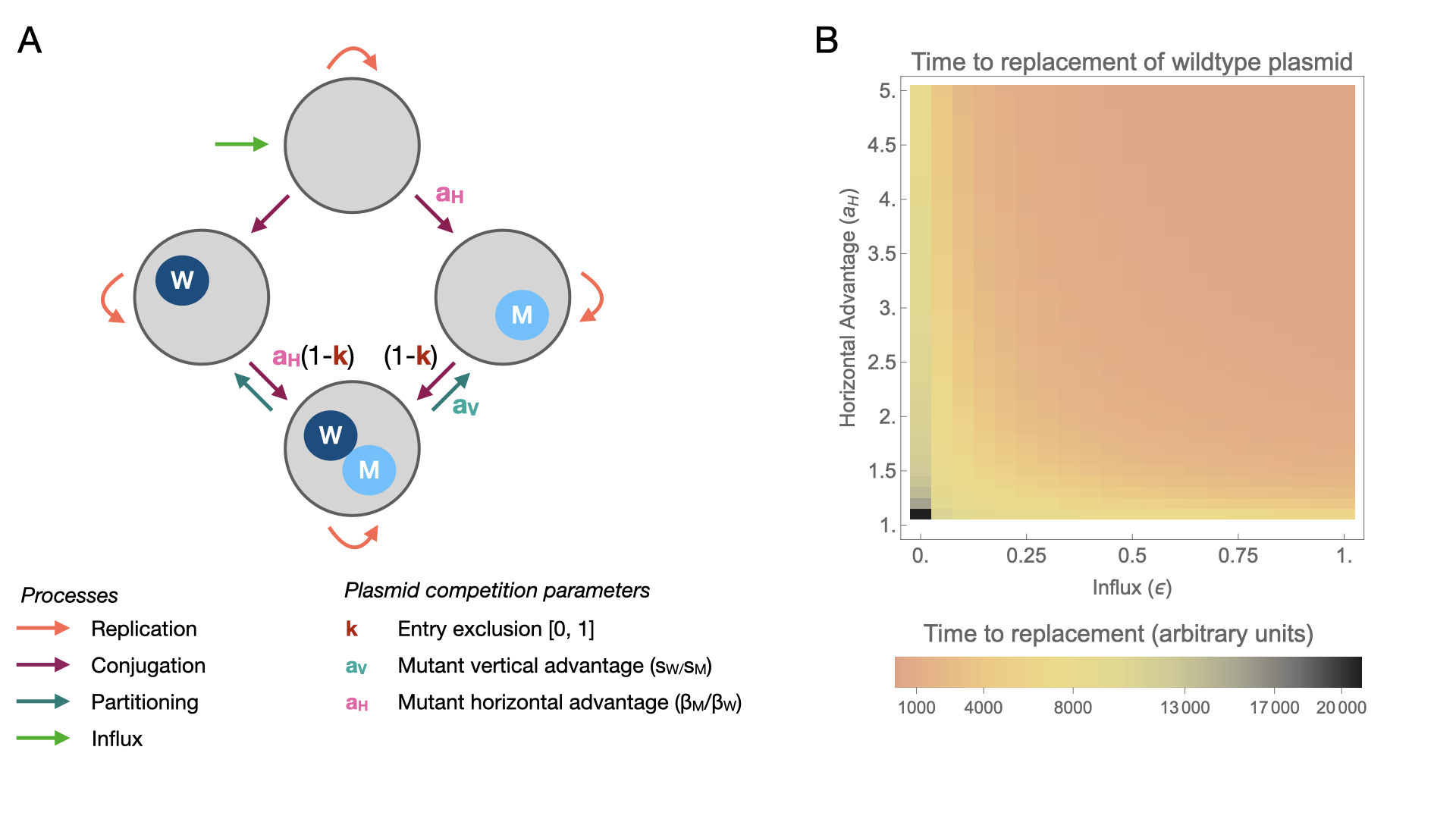

Supplement: S7 Fig — A. Schematic of the model, with the addition of an influx of plasmid-free cells (green arrow) in units of cells/(volume * time). The heatmaps shows the time (in arbitrary units) taken for the mutant plasmid to replace the wildtype plasmid (defined as the density of the wildtype falling below 0.01 cells/unit volume) as a function of horizontal and rate of influx of plasmid-free cells. Parameters values: ρ=1,c=110,γ=110,K=1,βW=1, sM=0.1, sW=0.1, k = 0.99, with βM=aHβW. We are interested in qualitative insights, the parameters are in arbitrary time units. White indicates no replacement. The data underlying this figure can be found in S2 Data. (TIFF) [file pbio.3003564.s010.tiff]

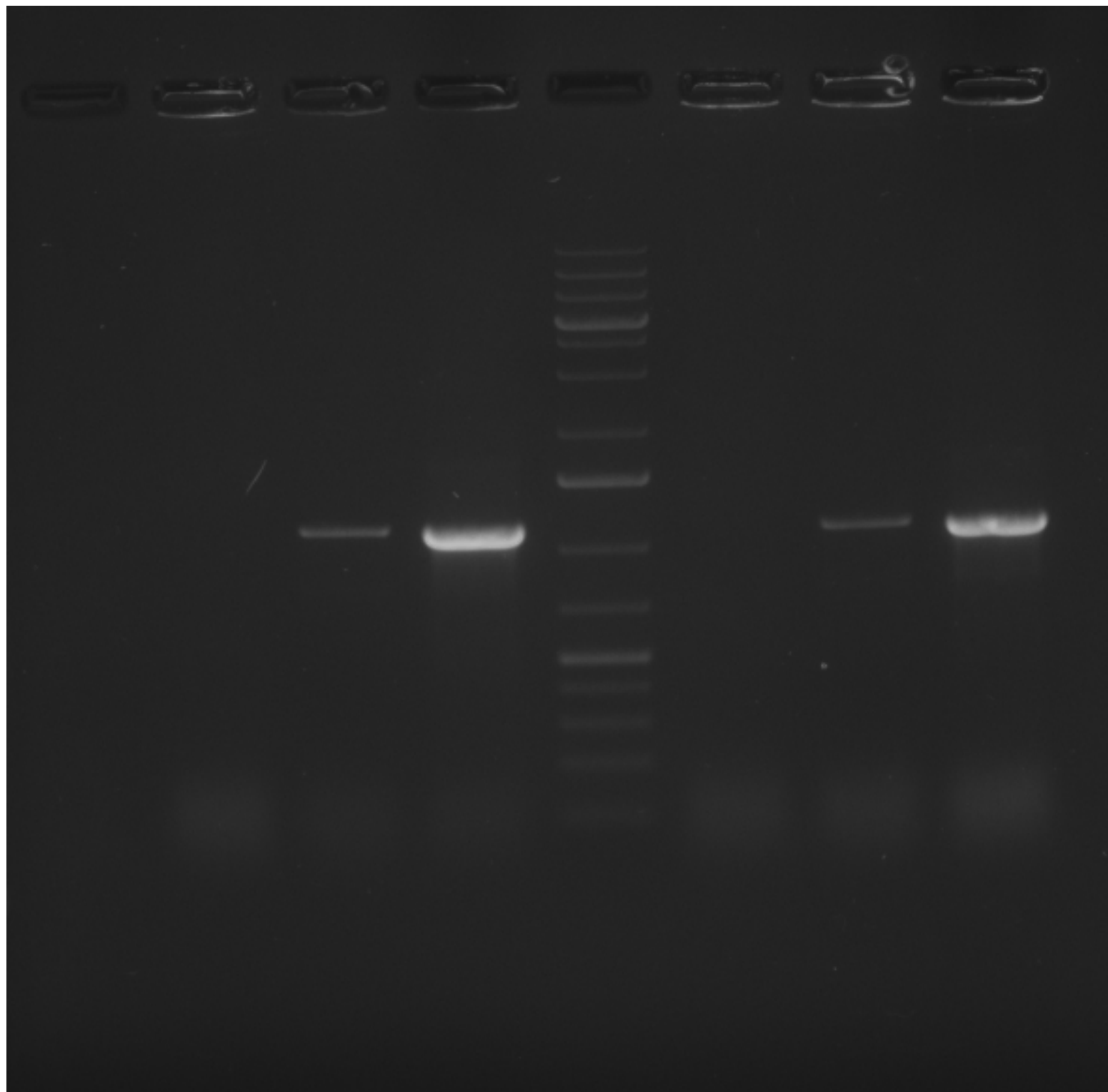

S2A

S2B

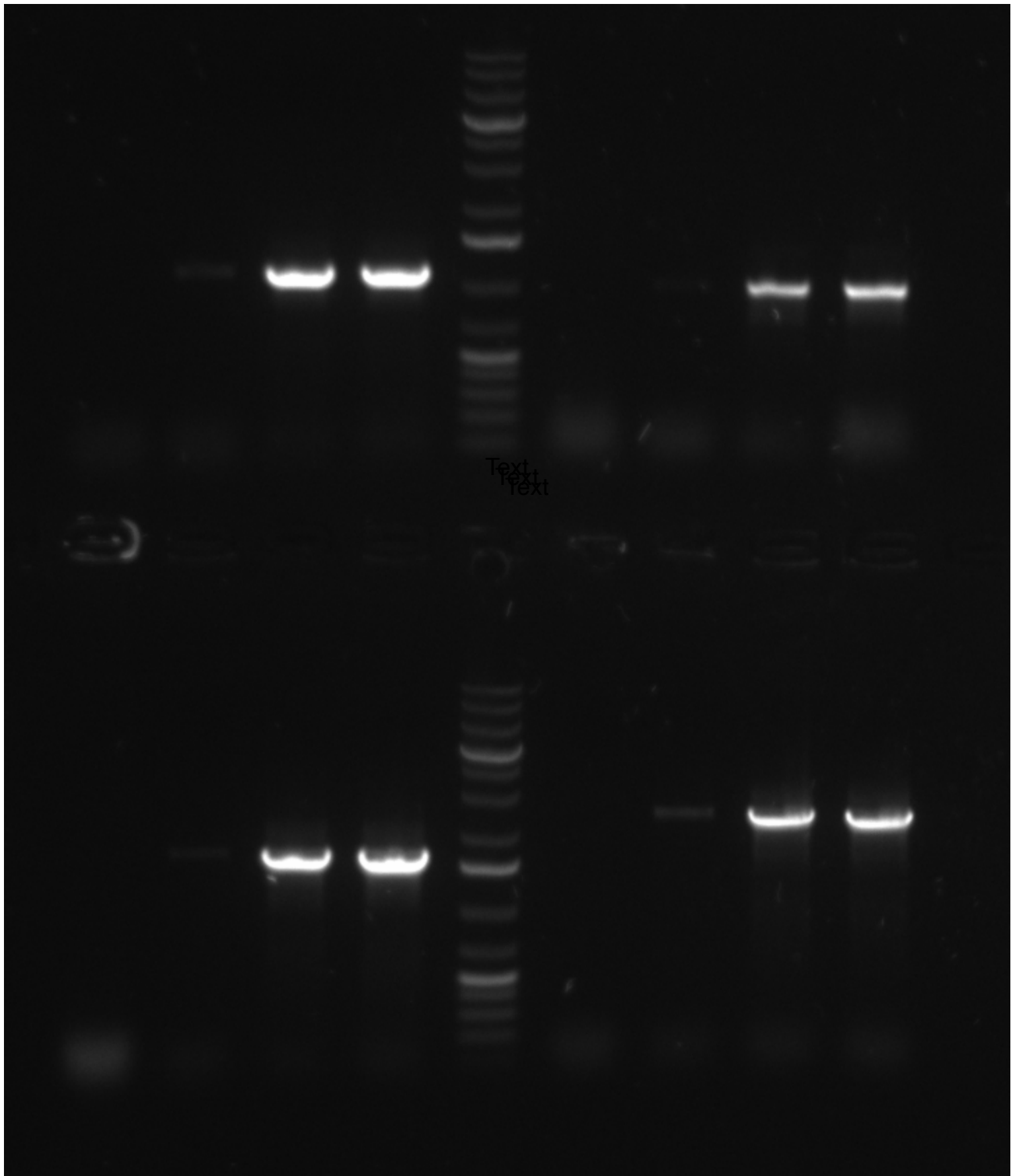

S2C

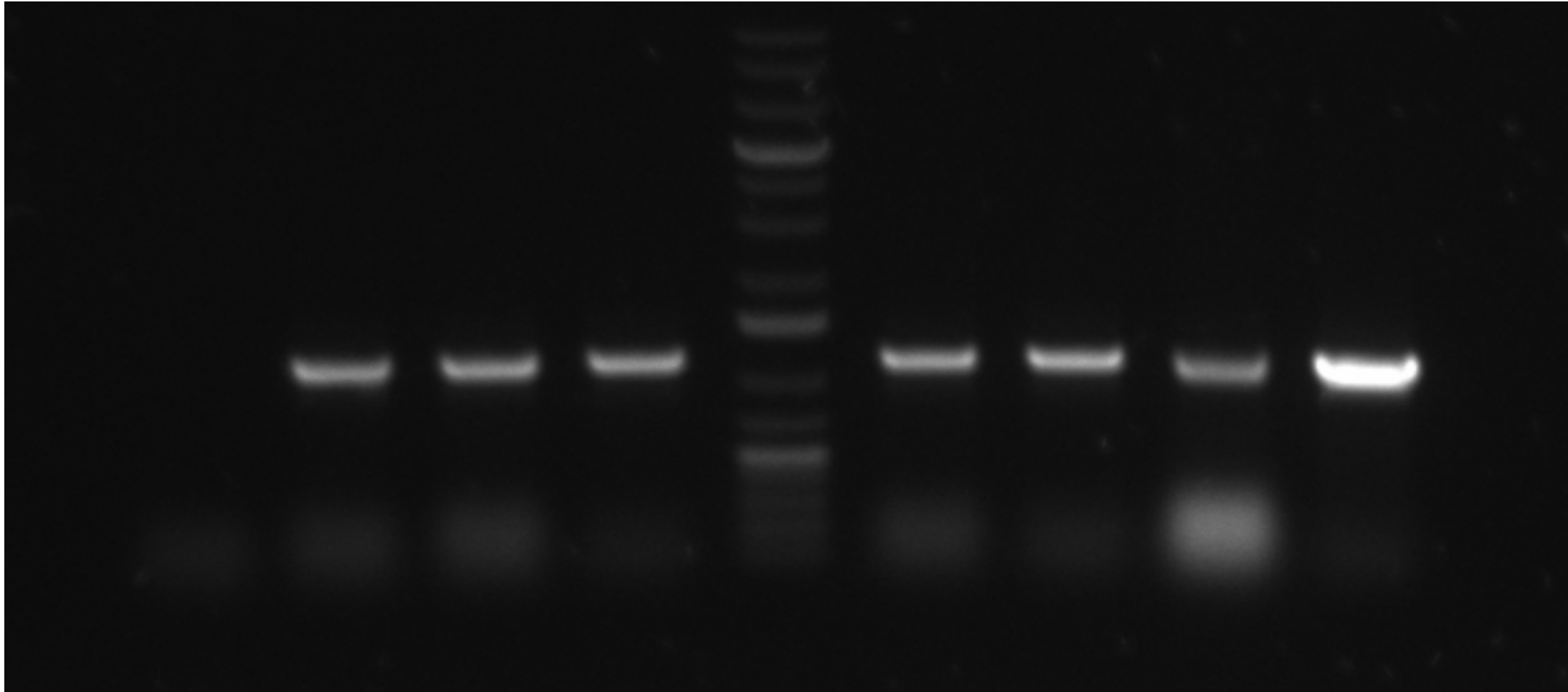

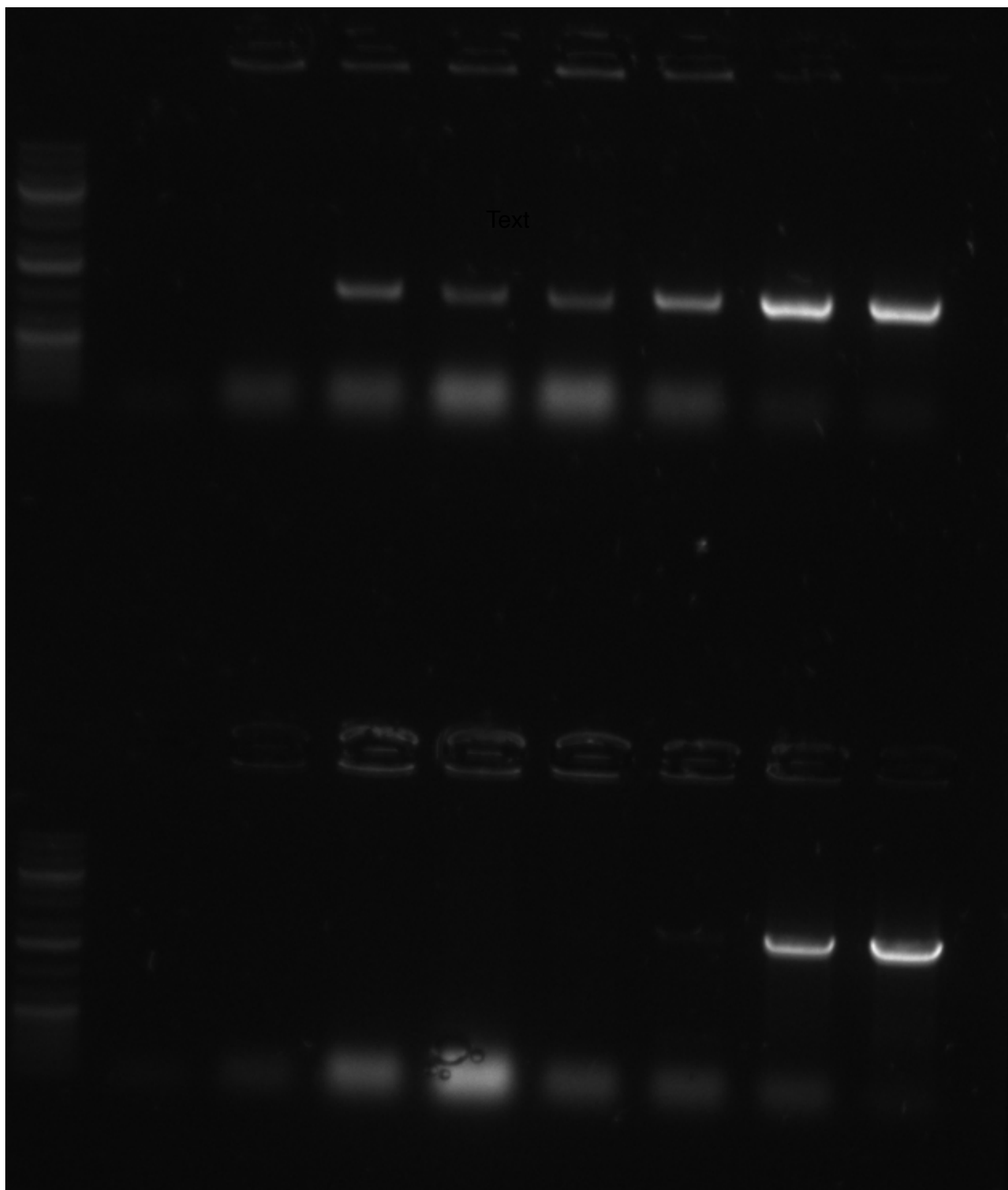

S4A

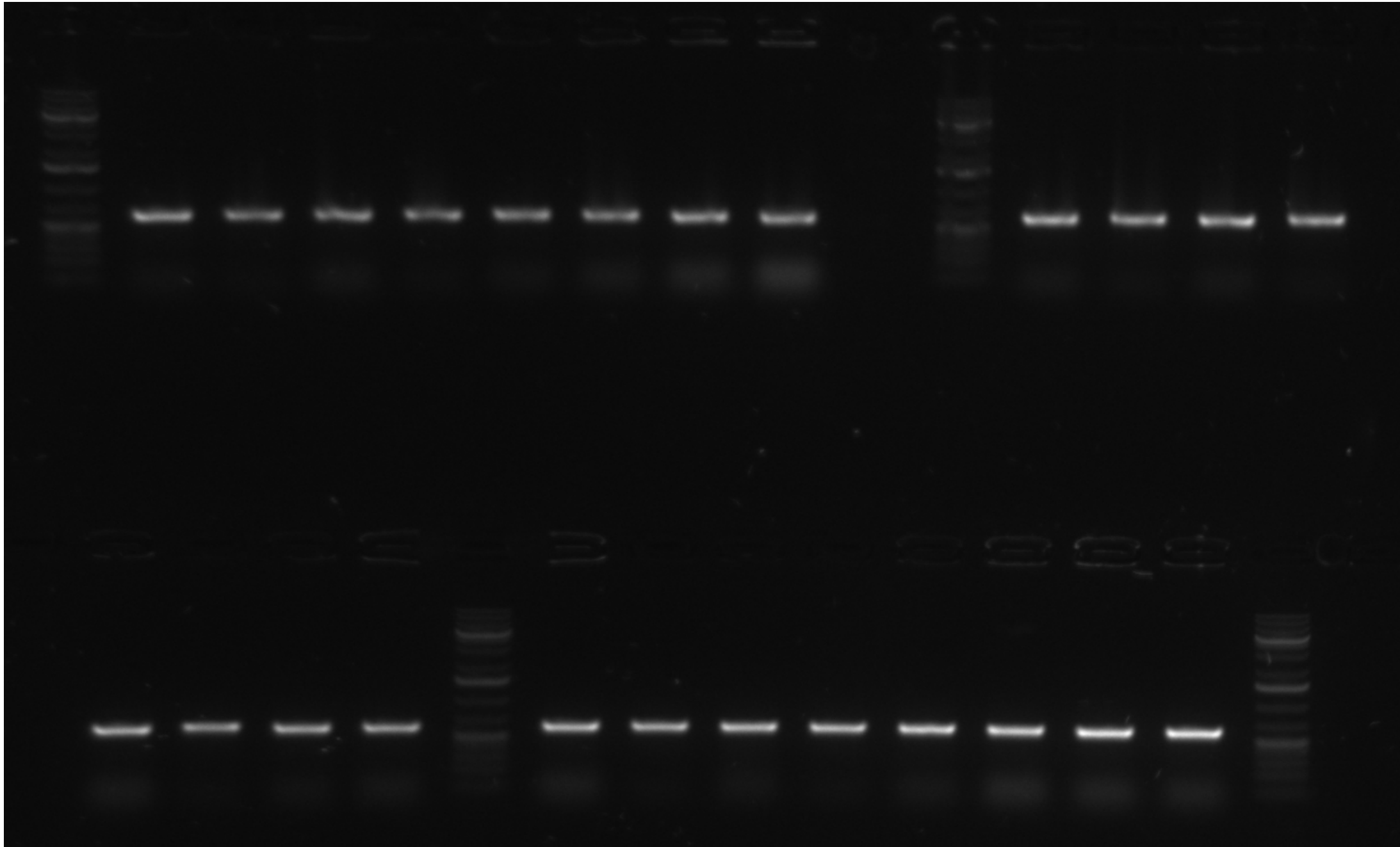

S4B

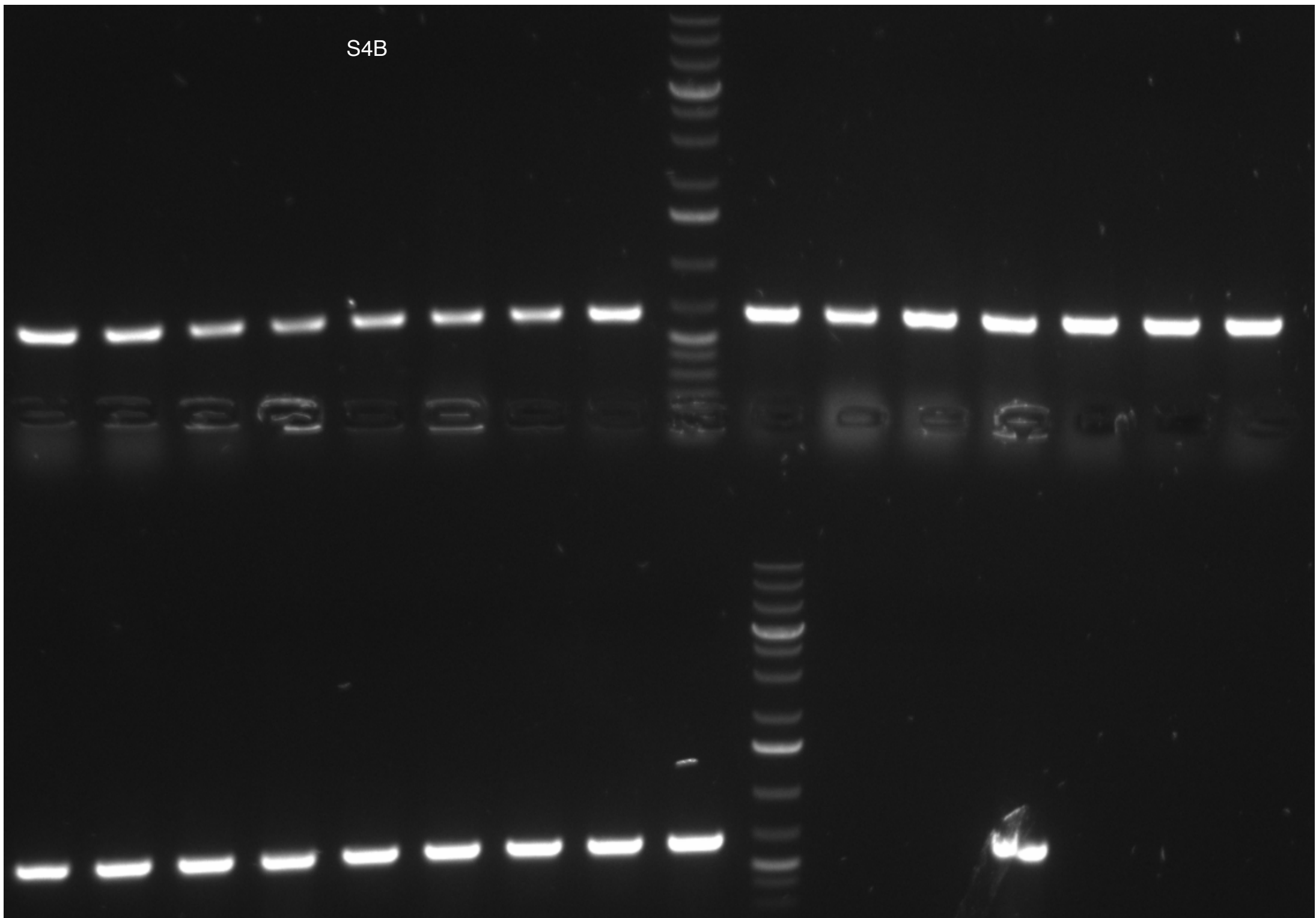

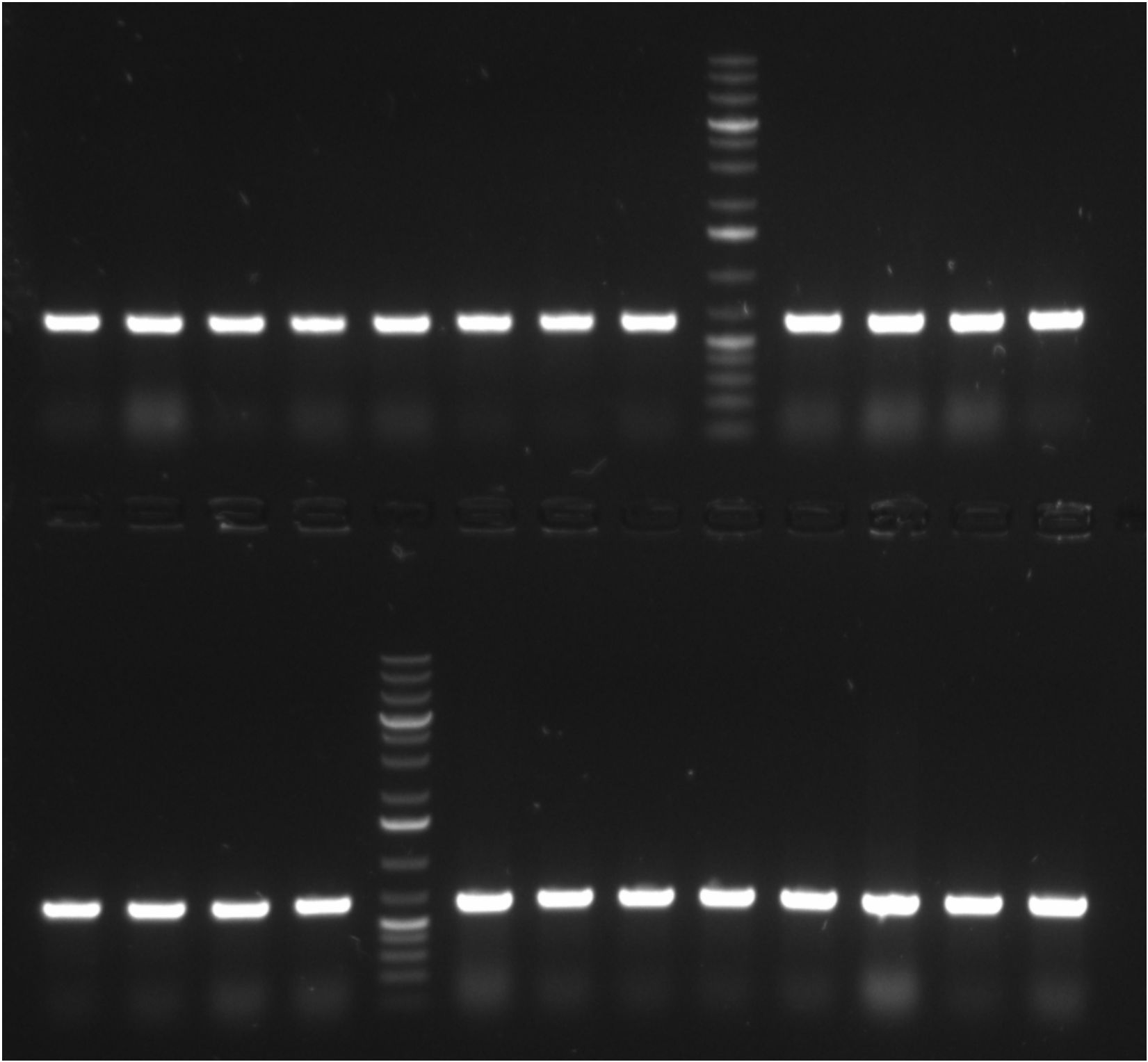

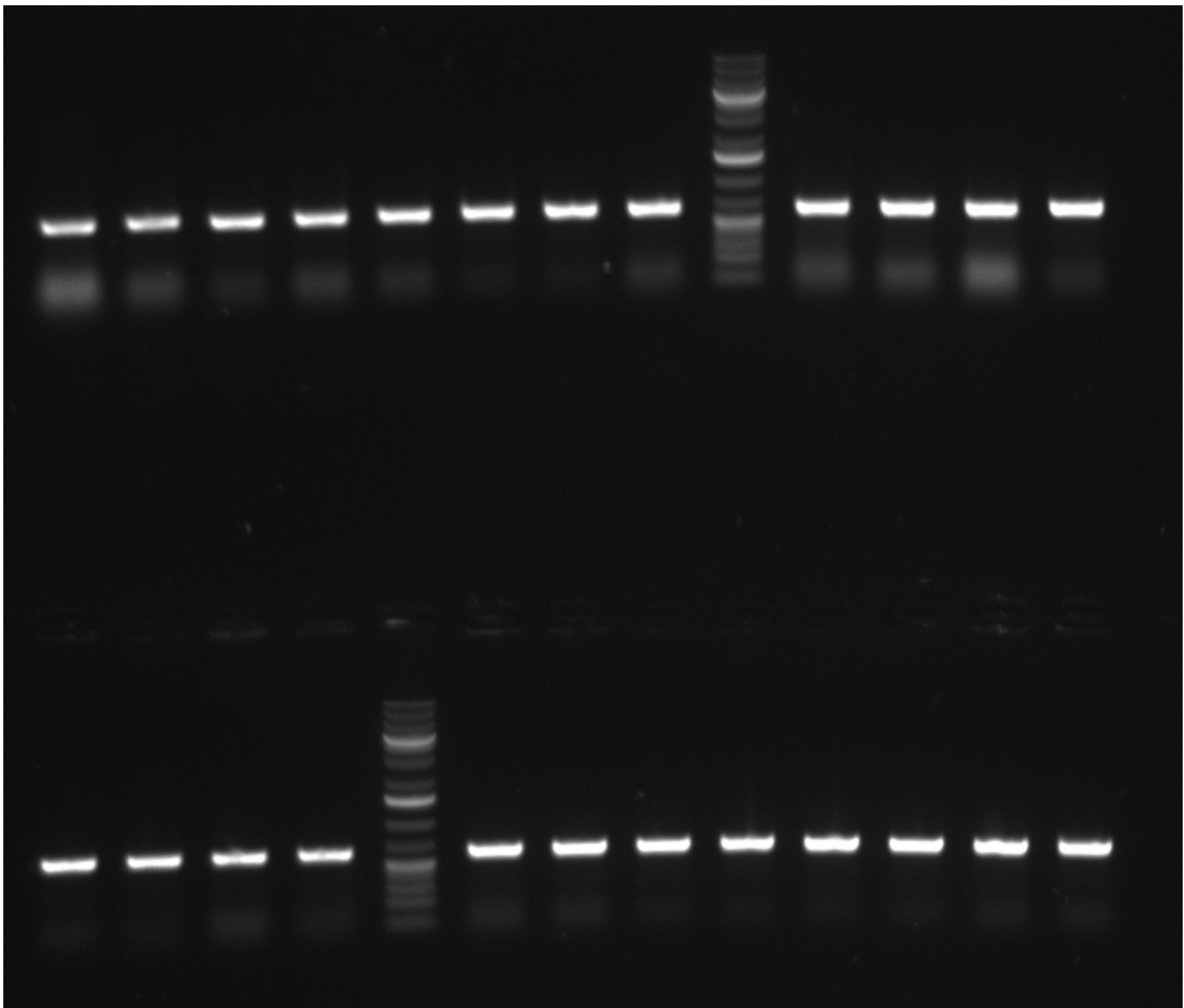

Supplement: S1 Raw Images. — Raw gel images associated with S2 and S4 Figs. (PDF) [file pbio.3003564.s013.pdf]
