# Supplementary material for: Plasmid streamlining drives the extinction of antibiotic resistance plasmids under selection for horizontal transmission
Source: PLoS Biol. 2025 Dec 11;23(12):e3003564. doi: 10.1371/journal.pbio.3003564 (PMC12711027; doi:10.1371/journal.pbio.3003564)
Supplement: Attachment — Submitted filename: response_to_reviewers_2.docx [file pbio.3003564.s016.docx]

As you can see, the reviewers think the work is interesting and well done, but they each raise important and overlapping concerns that should be addressed in the revision. Specifically, Reviewer #1 notes that additional data to support the generality of the importance of the plasmids in counteracting AMR is needed, including additional modelling and analyses of plasmid databases. In addition, the reviewers raise overlapping concerns with the accuracy and use of the term ‘stealth plasmid’ and note conceptual similarities with the findings of Zhang et al (PMID 31863068). We ask that the distinction between the current study and Zhang et al is discussed and clarified in the revised version. Finally, they raise concerns with the overall presentation of the manuscript and provide suggestions to improve the accessibility/readability of the article.

We thank the editor and all reviewers for very helpful comments. We have fully revised the manuscript to address these. Specifically, we have modified our vocabulary when discussing evolved plasmids, as we agree with the concerns on what the term “stealth” might imply. We have added a discussion of the similarities and distinctions with satellite plasmids. Finally, we have included a mathematical model that explores how a mutant plasmid with horizontal and/or vertical transmission advantages can displace an ancestral AMR plasmid, which allows us to better discuss the generality of our experimental results.

Our answers are shown in blue, with reviewer comments in black. Line numbers refer to the version with tracked changes; we have accepted some of the ‘deleted’ changes, mostly when they were replaced by other additions (these kept as tracked changes), to help with readability.

**Reviewer #1:** In this manuscript, Dimitriu and Matthews re-analyse samples from a previous experiment to investigate the evolution of plasmid-borne antimicrobial resistance. In that previous work, they identified that plasmid-borne antimicrobial resistance required the evolution of increased horizontal transmission and copy number in order to persist. In the current work, they unpick some of the underlying mechanism, and show the emergence of 'stealth' plasmids, lacking resistance genes, which could effectively outcompete resistance-encoding plasmids. This is an intriguing finding that meaningfully extends the previous work. The paper was clearly and engagingly written and presented. However, I found that several aspects of the current work could be better explained with reference to the previous study (oftentimes, elements of the current work seemed somewhat arbitrary to readers not familiar with the previous paper). I also think some further analyses could help generalise some of the findings further.

We thank the reviewer for their positive comments. We have aimed to explain and contextualise our work more clearly in reference to our previous study. We have performed several new analyses and included a mathematical model to generalise our findings.

Main comments:

- The main finding is that 'stealth plasmids', which do not confer AMR but are able to outcompete AMR plasmids in cells and in populations, emerge quickly by deletion and could help to block the transmission of AMR plasmids. This is an interesting finding, but it is hard to see how generalisable it is, i.e. to different plasmids or to natural populations. A couple of further analyses would extend the impact of the current work for the broader audiences of this journal. For example:

--> I wonder if it would be possible to adapt one of the well-developed mathematical models of plasmid dynamics to assess the conditions under which a stealth plasmid can prevent the maintenance of an AMR plasmid — I wonder if such a dynamic could be predicted, based on the extent to which the stealth plasmid impacts horizontal and vertical fitness?

--> What is the evidence for 'stealth plasmids' (i.e. cryptic plasmids with very high identity to sympatric AMR-encoding plasmids) in plasmid databases or in metagenomes?

I appreciate that these each would constitute a significant amount of work. However, though readers like myself would certainly be interested in the work in its current form, without some broader context, I am not sure whether the findings are sufficiently general for the wide readership of PLOS Biology.

We have followed the reviewer’s first suggestion and explored conditions in which ‘stealth’ (now streamlined) plasmids can displace an AMR plasmid, using mathematical modelling (new Fig. 7, new SI Figs. S6 and S7). We chose to analyse a simple model that provides qualitative insights and is generalisable, instead of precisely modelling our experimental system. The model shows that any horizontal or vertical transmission advantage would be enough for the mutant plasmid to ultimately displace the ancestral plasmid, and provides insights about some environmental conditions or plasmid properties that will accelerate or slow this displacement. We have used these insights to generalize our findings in the discussion, and discuss further the fact that even only an effect on horizontal transmission (as our data suggests that vertical transmission advantages might be less general across plasmid types) can be sufficient for AMR displacement.

In terms of bioinformatics evidence, we agree that evidence for stealth/streamlined plasmid existence in sequence databases would be very valuable. We thank the reviewer for spelling out very clearly what would be required: cryptic plasmids with high identity to sympatric AMR plasmids. This means that the data for such analysis would need to be high quality (either fully assembled, or at least high coverage data so that changes in coverage that can suggest deletions of AMR genes can be identified confidently), and at the same time have dense sampling within each ecosystem (so that we can detect diversity among sympatric plasmids). We are not aware of datasets that would be appropriate for this and believe that answering this question would require a specifically-designed future study.

- The finding has some conceptual resemblances to Zhang et al. (2019, doi: 10.1038/s41467-019-13709-x), which should be discussed.

We have added that reference and now discuss it lines 931-939.

Other comments/suggestions:

- It would be useful to give some wider context to R1. What kind of plasmid is it, are any similar plasmids involved in clinically important AMR? Etc. We have added some context when first introducing the plasmid, l 75-79.

- Line 73ff could be rewritten to better emphasise novelty/rationale of the current study. We have rewritten this for better emphasis (from l94).

- Line 75: define 'stealth plasmid', or put in inverted quotes. Is a 'stealth plasmid' the same as a cryptic plasmid? What is the difference with the 'satellite plasmids' of Zhang et al. 2019?

After considering this and other reviewer comments, we have changed the vocabulary we use to refer to these plasmids. We used ‘stealth’ based on their absence of phenotype when plating on antibiotics (so similar to a cryptic plasmid indeed), but this was not the main point we wish to make. As we now discuss lines 931-939, the main difference with satellite plasmids is that the plasmids we characterize still carry all their replication (and transfer) functions, and so do not behave as satellites but are fully autonomous.

- Line 88 ff. is in present tense. It is maybe a personal preference, but I would rather see all the results section in the past tense, describing specific work that has been done. We rewrote this paragraph in the past tense, and made some more changes throughout the results when describing specific experiments.

- Line 100. "Deleted plasmids" could imply the whole plasmid was deleted. Maybe "plasmid deletions"? Changed.

- Line 100ff describes contamination — how reliable is the rest of the experiment, in light of this observation? This finding could be explained a bit further for a wider audience, who may not understand the meaning of "nearby" (presumably this is an adjacent well in the 96-well plate?) We have added a few words to make this point clearer (this is indeed an adjacent well in the 96-well plate), and have added a reference (l157) to the supplementary text of our previous study, in which we discussed that contamination more extensively. We believe the rest of the experiment is still reliable for two reasons: i) sequencing data showed convergent evolution in terms of plasmid copy number evolution (different point mutations which thus evolved independently and were not due to a single event followed by contamination), and ii) for the current study, we independently repeated a simplified version of the full experiment, and observed similar plasmid population dynamics and evolution (Fig. 2).

- Line 109. I feel more could be done to get to the bottom of the faint band observed in the ancestral R1 plasmid. Does this occur in colony PCR? Does it occur with other primer designs? Is it possible to estimate the rate of excision, e.g. through use of qPCR?

We have performed additional experiments to characterise this (shown in new Fig. S2 and discussed l 168-176). This faint band does occur in colony PCR (all our experiments were done using colony PCR), and does occur with three other primer designs we tested. For all primer pairs, the faint band is of the same size as the band predicted, and observed, in the variants with the full deletion; and sequencing the faint band obtained with the original primer pair showed the exact same sequence as expected for the full deletion, thus we do not believe this is some simple non-specific binding. However, using a primer pair further away from the deletion, together with increasing annealing temperature, this faint band disappears. Moreover, the presence of the band was not affected when we transferred R1 to a *recA* knock-out strain background, which is deficient in homologous recombination. For this reason, we believe now that that band is due to some kind of *in vitro* artefact e.g. the polymerase switching template when encountering the IS repeated region, which is linked to the presence of repeated ISs in the wildtype plasmid, but is sensitive to PCR conditions and not due to recombination *in vivo*.

- Fig. 2. What does 'mut' refer to? I understand this is discussed in the methods section and in the previous paper. I think a bit more could be done to make the current work stand more independently, by briefly explaining the justification for this design. We have now added a brief explanation of the experimental design and nomenclature used at the start of the results section, and within Figure 1A, and justify the use of the ‘mut’ strain in the methods.

- Line 210. Typo: "extent" – thank you – fixed.

- Line 212. Which stealth mutant is a finO derepressed mutant? This could be indicated here, when the concept is first introduced. It is mutant s_m68c_. We have re-ordered the results section, and now explain the *finO* mutation when first introduced (l 274-278), and again when specifically mentioning s_m68c_ (l 314).

- Line 213. "Expect this to impact horizontal transmission" — what exactly? The copA mutations or finO? Clarify. We meant finO there but agree this was unclear. We have explained that both mutations should impact transmission and why, in a new paragraph (l272-275).

- Line 248. The rationale for testing with ∆lac and RifR is not made clear. Which was used in the evolution experiments? Again, a few words could explain. Why are there gaps, i.e. why were some plasmids only tested in some hosts, and not both? Perhaps it would be better to reorient the subplots in Fig. 5 so they are placed horizontally, which would allow you to drop values from the x-axes that do not apply (e.g. sm97f/RifR), and enable better comparisons on the y-axis across all strains. We used these two strains because they were used for different transmission experiments. We have made this clear l286 and introduced all strains used better in the Methods section (l630-632). The gaps in our initial dataset were due to the fact that we had not obtained all plasmids in both strains at the time; we have obtained a full dataset since and now present results for each plasmid in both hosts (now Fig. 3). We could possibly have focused on only one strain as qualitative results are similar for both, and this would arguably be easier to discuss, but we did observe some quantitative difference in the costs between strains and felt it would be disingenuous to then arbitrarily exclude half of the results for convenience.

- Line 252. Formatting of p-values needs correction (there is a 'times' symbol missing). Thank you, we have fixed this throughout the text.

- Fig 5 is not consistent with text. Figure shows sw90d and sm97f as orange points with red bars, which according to the legend describes plasmids that have repressed transfer and high PCN. However, in the text these plasmids are referred to as "low PCN stealth variants" (line 265). This was an error in the text which we have corrected, these plasmids have high PCN. We also changed the way we present the results, in response to another reviewer’s comments, as the color legend was confusing.

- Section beginning line 246 concludes that cost to the host is determined by transfer gene expression. This finding is drawn from the observation that plasmids with derepressed transfer (finO, sm68c) are more costly. However, we don't actually know what the transfer rate (or indeed transfer gene expression) is in these clones, only that they invade populations faster than the ancestral plasmids. We have data on transfer rate for the AMR R1*finO*, from our previous publication and also now included in Fig. 6A (~10^4^ increase in transfer when measured in an 1h transfer assay). But we agree we do not have such data for s_m68c_, and we have rewritten that results section to be more careful in our wording and focus on genotypes more than assumed phenotypes.

- What threshold is used to define 'high PCN' and 'low PCN', and why? For Fig. 5 (now Fig. 3), we were also using genotype information (presence of copA* mutations) for this, so have replaced “high PCN” and “low PCN” by copA* mutation presence. copA* mutants do have a reliably higher PCN, with all clones with copA* mutation frequency >0.9 having a relative coverage of the plasmid backbone compared to the ancestor > 14 in sequence data, and all clones with no copA* mutation a relative coverage < 10 (see data associated with Fig. S3).

- Fig 6 was a little confusing — it was hard to see the points for the stealth plasmids in the left hand sub panel of panel B. Perhaps if you include the limit of detection on the y-axis , then these points could be shown more visibly with the connecting lines. We have performed additional experiments which now replace Fig. 6 and solve this limit of detection problem (using selective markers to distinguish between donors and recipients, instead of relying on colony color as previously, which strongly limited our power of detection).

- Section beginning line 287 is interesting and I like how you contextualise the findings in a broader context of MGE competition. As discussed above, it would be exciting to generalise this further e.g. using a mathematical model, and/or analysis of published sequences.

We thank the reviewer for their appreciation of our findings and believe that the mathematical model allows for some generalisation of our results. Ultimately, we will aim in the future to explore how these results are generalisable, to other systems experimentally, and in natural environments.

**Reviewer #2:** The manuscript 'Stealth Plasmids: Rapid Evolution of Deleted Plasmids Can Displace Antibiotic Resistance Plasmids Under Selection for Horizontal Transmission' by Matthews & Dimitriu is both interesting and highly relevant for researchers working in bacteriology, plasmid biology, antibiotic resistance, and molecular evolution. I have several major and minor comments that I hope the authors can use to improve the manuscript.

We thank the reviewer for their comments and believe we have improved the manuscript significantly in response to them.

Comments/Concerns:

1.	Readability:

a. While I understand the authors' desire to reference and discuss their previous work (PNAS 2021), this section makes the manuscript slightly tedious to read in the beginning. Could the authors consider limiting the discussion of the 2021 paper to a few key points and moving the detailed discussion to the Discussion section?

b. Regarding the above, is the lower part of Figure 1A necessary?

We agree with the reviewer and have reformatted our introduction and early results section aiming to focus on the key points most relevant to the description of our new results. This includes removing gene dosage discussions that were not required here (l89), but also adding some clarification of the nomenclature we use instead. We have removed the lower part of Fig. 1A.

c. Line 196: The manuscript refers to Fig. 3B, but this figure does not appear. Fixed – we meant Fig. 3 (now Fig. 4).

d. I do not think the term "Stealth Plasmids" is appropriate for the modified plasmids. While these plasmids may have appeared 'stealth' from the perspective of the PNAS 2021 paper authors, the term does not convey the correct meaning for readers of this manuscript. It implies that the plasmids lose antimicrobial resistance (AMR) as a strategy, which is not the case.

We thank the reviewer for this comment and pointing out clearly what the issue is. Indeed we used ‘stealth’ from our point of view as experimenters (and will likely continue to do so in the lab when doing experiments that involve selective plating on antibiotics!), but the behaviour of these plasmids in terms of antibiotic resistance is not relevant to competition in these experiments performed in the absence of antibiotics. Instead, we are now referring to these as ‘streamlined’ plasmids, which we hope conveys better the fact that they are stripped of unnecessary functions. We have modified the text throughout.

e. I also disagree with the use of the term "deleted plasmids." To me, this implies that the plasmids are completely absent from the cell, when in fact they are present but have portions of their sequence deleted. We have similarly removed references to ‘deleted plasmids’ throughout the manuscript.

f. The names of the strains were difficult to follow and understand until the Methods section (which appears after the Discussion only). In the Methods section, these names are well explained (lines 426-428). I suggest that the strain names be introduced earlier, preferably in the Results section. We have added a ‘clone nomenclature’ summary within Figure 1A and referenced it at the start of the Results section.

g. While reading the Results section, I assumed that "mut" referred to "mutator." This is only clarified in the Methods section. We now mention this at the start of the Results section (l109).

h. For Figures 2B and 4B, the horizontal axes appear to represent frequency (ranging from 0 to 1), not percentages (0 to 100), correct? Thank you, we have made the change.

i. I think Fig. 5 extremely difficult to read. Moreover, I think the legend is wrong at "low PCN and high PCN": I think dots should be there, not boxes. We have changed how we present the results of Fig. 5 (now Fig. 3), in addition to now having a full set of data in the two strains we used to measure plasmid cost. We now indicate key properties of each plasmid above the graph instead of representing it with colors.

j. The vertical axis of Fig 5 is h^^-1^, not h-1. On the right of Fig.5 dlac should delta-lac. Thank you, we have made these changes and fixed other symbol / typography issues in all figures.

k. Line 268: missing "x" in p=6.8x10-6 and in line 271 p=3.7x10-5. The same in line 279 and possibly other places Thank you, we have made these changes throughout.

L. line 293 "include 3…" should be "include three…". The same in line 308 Fixed.

m. 1st paragraph of Discussion needs the reference to PNAS 2021 paper. Added.

2. In the Results section, it was unclear where the RM genes were located. I initially thought they might be on the R1 plasmid (or in the mutant plasmids), or in the chromosome. However, it turns out they are on artificial non-conjugative plasmids (pEcorRI and pEcoRV, as explained in the Methods section). I think this should be briefly mentioned in the Results section. Could the authors discuss how the location of these RM systems on plasmids might influence the results? We have made explicit in the results that the RM systems are located on small cloning plasmids (l420). We have also added a sentence in the discussion to mention that they might be expressed at higher levels than in natural isolates (l592-595).

3. The pEcorRI and pEcoRV plasmids were "maintained using their ampicillin resistance marker" (line 423). When exactly was the ampicillin used? Was it just before the experiments in Fig. 5, or was it used during the 3 days mentioned in Fig. 5? This could potentially affect the results. It was only before the experiments as we did not want to introduce another variation between treatments during the experiments. Thank you for noticing this, we have added the information in the methods (l650). Anecdotally, in a separate experiment that ran over 11 days we did not observe any loss of these plasmids and we believe these plasmids do are stable in the absence of antibiotics as RM systems can act as addiction systems.

4. I am concerned that in several experiments, the authors concluded that the AMR region was absent simply because plasmid-bearing cells were ampicillin-sensitive. However, the plasmids could still carry resistance genes for the other antibiotics typically carried by the R1 plasmid (e.g., sulfonamides, streptomycin, spectinomycin, kanamycin, chloramphenicol, and, of course, ampicillin). Figure 1C suggests that losing ampicillin resistance (AmpR) implies losing all resistance markers, but this is not certain, is it?

We agree that this is technically correct, however Fig 1C suggests that the loss of the full AMR region is by far the most common event. Moreover, in our experiments we are either following specifically the plasmids with full deletion (Fig. 2 and Fig. 5C, using PCR with R1_del_ primers, which would not yield a band of the expected size if some other resistance genes were still present), or focusing on the ecological dynamics of the AMR plasmid at short timescales (< 8 days), for which a new stability experiment (Fig. S4) shows that no loss of AMR is detected.

**Reviewer #3:** The authors of this study follow up on the results found in a previous study, which found that antimicrobial resistance encoded on conjugal plasmids was lost from the population under regular recipient invasion (Dimitriu et al., 2021). In their recent study the authors add another condition to their former results and investigate the part of the evolved population, that did retain the plasmid backbone, but lost the antimicrobial resistance genes due to a recombination event between two ISs on R1. Their experiments suggest an advantage in vertical and horizontal transfer due to multiple factors like increased plasmid copy number by comparing several stealth plasmid variants and the ancestral R1. Furthermore, they show that stealth plasmids do provide a more efficient barrier against incoming related plasmids compared to restriction-modification-systems.

Major comment

I find the term 'stealth plasmid' inaccurate - that R1 plasmid variant is lacking an AMR island, but is still able to replicate and conjugate (not as e.g., described in Zhang et al. 2019 Nat Commun). So, in essence, the authors are following here the dynamics of several R1 variants. We thank the reviewer for the comment, and have modified how we call these plasmids, in response to this and other reviewers comments. We used ‘stealth’ from our point of view as experimenters, but this did not describe well how they behave within the initial evolution experiment. We are now referring to these as ‘streamlined’ plasmids, which we hope conveys better the fact that they are stripped of unnecessary functions. We have also added a discussion of how these plasmids compare to the satellite plasmids of Zhang et al (lines 931-939).

The specific experiments are well designed and rather easy to follow. However, as mentioned below, quite few controls seem to be missing, which makes it hard to evaluate its true relevance i.e. evolution of stealth plasmids. Nevertheless, the authors give an example of the complexity of factors like plasmid copy number and gene expression influence plasmid success in a naturally evolved plasmid population.

We thank the reviewer for their comments. We have added several experiments and controls, which we believe make our results more robust.

I would find the manuscript overall somewhat easier to read if the experimental system would be presented at the results beginning with more detail. I understand that author's wish to connect this study to the previous manuscript, at the same time, it seems that they expect some prior knowledge of that work while reading the current study. A clearer presentation of the different plasmids and hosts in the beginning would help to understand the overall results much better.

We have reformulated both the end of the introduction and the start of the results section, and hope that it now reads better. We have also clarified the nomenclature we use to refer to the variant plasmids in Fig. 1A, and introduced both strains and plasmids more explicitly.

Other general comments

General comment about the strain/clone naming: In line 102 the authors call one clone w_e, however afterwards they refer to it without further explanation by sw_e. I assume, that w_e and similar denominations refer to the plasmid itself, and sxyz to the bacteria carrying it. It would be helpful to make this clear in some part of the manuscript. Thank you for the comment, we realize now we never made this explicit. We meant the opposite (w_e referring to the isolated bacterial clone, s_w_e_ to a specific plasmid variant), and have now stated this in the text (l 153-157) and provide an example in Fig 1C for clarity.

General comment about the figures displaying CFU/ml: I personally prefer the display of frequencies within the whole population or the recipient population, since life titer or recipient CFU/ml can change slightly daily and it is easier to comprehend the rarity of changes the population undergoes. In my opinion this would add another layer of understanding to the data graphs.

We agree that in most cases frequencies are a better readout of the biologically interesting dynamics than CFU/mL, when available. We have made the change for Fig 3 (now Fig 4) and Fig 4 (now Fig 5). We have kept CFU/mL data for Fig 2 as we did not quantify the whole population for all timepoints in experiment 1 so are unable to calculate frequencies of the AMR population. In Fig. 6B (new timecourse data similar to previous Fig. 6) we have kept transconjugant density to avoid what we believe are uninformative threshold effects – in some treatments we have 0 colonies at early timepoints, and calculate a threshold density of transconjugants corresponding to the limit of detection. Because of the growth dynamics of recipient cells, this would translate into an initial decrease in the ‘threshold proportion’ of transconjugants within the recipient population that we are able to detect, which does not correspond to a real decrease.

Comments on text and figures

Line 96-99: The seeding cell of the colony retained from the experiment must have harbored a mixture of R1 variants, I agree. However, resistance to ampicillin by bla is provided by secretion of the gene product. Therefore, the assumption of the plasmid variants co-existing within each bacterial clone is at least unprecise because segregational drift likely has led to homoplasmic daughter cells carrying one of either variants.

By clone, we meant “bacterial population descended from a single colony” here. But we appreciate that “clone” can be interpreted in different ways, and have replaced this by “sequenced bacterial population” (l141). We also fully agree with the reviewer that our results might have looked very different (and we might have missed the story described here for much longer) if we had selected clones using a different antibiotic for which resistance is not a public good.

Line 109 - 111: Considering, that the authors base their latter detection method on the primer pair used here, I would like to see a deeper analysis of the faint band detected in the ancestral R1 carrying clone. This could either involve the sequencing of the PCR product or designing another primer set to validate that this is no PCR artefact from unspecific priming. We have performed additional experiments to characterise this (shown in new Fig. S2 and results l168-176). This faint band does occur with three other primer designs. For all primer pairs, the faint band is of the same size as the band predicted and observed in the variants with the full deletion; and sequencing the faint band obtained with the original primer pair showed the exact same sequence as expected for the full deletion (we had actually sequenced this band at the same time as the band from s_w_e_, but had forgotten to specify this in our initial submission, we spell it out in the results now). For this reason, we do not believe this is some simple non-specific binding. However, using the primer pair further away from the deletion, and increasing annealing temperature, this faint band disappears. Moreover, the presence of the band was not affected when we transferred R1 to a *recA* knock-out strain background, which is deficient in homologous recombination. For this reason, we believe that that band is due to some kind of *in vitro* artefact e.g. the polymerase switching template when encountering the IS repeated region, which is linked to the presence of repeated ISs in the wildtype plasmid, but is sensitive to PCR conditions and not due to recombination within colonies happening *in vivo*.

Also, this faint band raises the question when colonies in latter experiments analyzed by colony PCR were considered carrying R1del. Colony PCR experiments with R1_del_ primers always included a negative control (R1-carrying colony) and a positive control (s_w_e_-carrying colony) so we could visualise clearly which bands were faint or strong. The distinction between both types of bands was always clear, and the controls were really useful only when only one type of band was present in the tested samples. We have now made that explicit in the results (l175-176) and methods (l678-680) sections.

Line 136 - 142: I agree with the general conclusion that likely the Rdel variants also have a copA* allele encoded on them, while the AMR encoding plasmids still have the ancestral PCN. However, the combination of more variants (derived from homologous recombination between the two) could also be possible. The authors could consolidate their assumption by investigating the coverage of the copA* alleles vs. the coverage of copAWT in the same clone, then comparing it to the frequencies of the AMR coverage. We thank the reviewer for this suggestion and now provide that graph as a new version of Fig. S3B – indeed the frequency of the AMR coverage is negatively, linearly correlated to the relative coverage of the copA* alleles.

Figure 2: In Figure S2 the authors show that the strain carrying R1WT produces a faint band for the R1del primer pair as well. I assume the same strain has been used to start the evolution experiments. Therefore, showing the original R1del frequency in colonies similar to Fig. 2B or even better determining it by qPCR/ddPCR would allow for interpretation if new Rdel arose or if they have been already present in the starting culture. If latter is true, additionally segregational drift needs to be taken into account (assuming co-existence of plasmid variants in one cell) as well as fitness of homoplasmic carriers of one or the other variant (which would be best to be provided in any case, if possible). Unless this is cleared, I do not agree with the interpretation of "stealth plasmids evolve" (line 182).

Moreover, this analysis of t0 clones could also explain the differences between experiment 1 and experiment 2 (since the composition of plasmid variants could be different for the two starting cultures).

We agree that there will likely have been differences between starting cultures that might have contributed to the different dynamics of plasmid variants. However, based on our new PCR data (Fig. S2) we believe that the faint band observed by PCR is not an accurate read-out of the plasmid variant composition within the colonies tested, but instead an *in vitro* phenomenon. More generally, we agree with the reviewer that segregational drift and fitness of plasmid hosts should ideally all be characterised, but this would require better tools than what we have available for this study.

As we cannot rule out that the variant plasmids were already present in the starting cultures, we have reformulated the statement pointed out above, and now state “instead, streamlined variant plasmids spread through populations” (l252).

Line 190 following:

The results presented in Fig. 3 are tricky to interpret - the authors should supply data on plasmid stability (or segregational loss) in the form of % hosts in the population rather than frequency alone.

As detailed above, we now present the data for this figure (Fig. 4 now) as proportion of hosts carrying AmpR in the population. We have also modified the x axis after realising the time scale was not linear in our previous graph.

Furthermore, I cannot comprehend from the manuscript how the authors made sure their competition experiments were not influenced by horizontal transmission. Or at least that there is no plasmid loss during culturing, then it could be assumed that surface exclusion is preventing horizontal transmission. Since the authors show later, that transferring faster than R1, plasmid-free cells arising during the experiment could falsify results. In my opinion, either inhibition of conjugation e.g. by linolic acid (Ripoll-Rozada et al., 2016) or a stability experiment showing no substantial plasmid loss would be needed.

We had indeed been assuming that surface exclusion is preventing significant rates of horizontal transmission in the population at this time scale, due to all hosts initially carrying plasmids and R1 plasmid carrying stability systems that limit plasmid loss. We had also chosen a maximal timescale of 8 days for competition experiments based on our evolution experiments results, which showed no substantial decline in AMR density in two independent experiments. To confirm this, we have now carried out a plasmid stability experiment over 8 days, using some of the main variants we studied (R1, s_w_e_ and s_w90d_). We detected no plasmid loss, showing that plasmid loss is not substantial at the timescale of these competition experiments. The added R1_TcR_ control in Fig. 6 (see below) also further suggests that plasmid carriage indeed strongly limits horizontal transmission of an incoming plasmid (500- to 1000-fold effect at 24h in Fig. 6B).

Line 196: There is no Figure 3B. Fixed – this referred to Fig 3.

Line 207: Please add details on the design of that evolution experiment (beyond the caption of figure 4). Is that the same design as presented in Fig. 1A? What is the daily mating/ transfer regime? There was no influx of cells after the first day here, we added some details to make that clear (l356).

Figure 4 and respective paragraphs: Since the horizontal spread of the plasmid over a prolonged time, bacterial growth i.e. inter-cellular competition could have an effect (as already observed for sw90d in Figure 3) as well as varying plasmid acquisition costs between R1 variants (see (Ahmad et al., 2023)). This could be avoided by conducting the mating experiment and recording transconjugant frequencies already after a short time interval, that allowed for only 0-3 divisions would be more insightful.

We would have liked to do this experiment but are limited by the absence of selective markers on the variants, and screening of transconjugants by PCR of recipient cells is not feasible at this timescale: as an example, results shown in the new Fig. 6A from 1h transfer assays (in which we could limit inter-cellular competition and acquisition costs), correspond for R1wt to a proportion of recipient cells which acquired the plasmid which is below 10^-4^, so we would have had to screen an unfeasibly high number of colonies to get meaningful data. We agree that both cell competition and acquisition cost will also have an effect, but designed that experiment to make horizontal transmission as central to plasmid population dynamics as possible in a relatively short-term experiment. We have added more discussion of these other confounding factors in the discussion (l531-536).

Also - the figure will be easier to read with a proper legend (indeed, C is almost informative but 'none' = black? is 0 and hence not shown). A simple graphic illustration of the plasmid variants would be also very helpful. We have added that legend in Fig. 4 (now Fig. 5), as well as a summary of *copA** or *finO* mutation presence on the variants.

Line 246: please elaborate on the motivation for this section. Is it possible that the results observed in Fig. 4 are due to fitness differences between hosts of the different plasmid variants? Answering that may require head-to-head competitions rather than growth rate comparisons.

We have re-ordered the results section since adding the new stability data (Fig. S4), and now present data relating to vertical transmission (costs, then within-cell competition) before data relating to horizontal transmission. We introduce the growth rate data (now Fig. 3) as a way to estimate the effect of plasmids on host growth, We agree that fitness assays with head-to-head competitions would be ultimately better, but privileged growth rate measurements as they were more achievable for the relatively high number of plasmid variants we wanted to compare.

In terms of impact on Fig. 4 (now 5), as mentioned above, we are now explicitly discussing fitness and acquisition costs when discussing these results in the discussion.

Also - it would be easier to follow if the authors explain the two strains (MG ∆lac and MG RifR) earlier in the manuscript (I could not easily identify where are those coming from).

We now briefly introduce the strains in the results (l286), and detail better all strains used in the methods section (l631-633).

Line 283-285 - this conclusion seems to me as going beyond what can be interpreted from the comparison of growth rates without further validation, especially the AMR region deletion relieving the fitness cost. Such an assertion should be tested, eg, by replacing the AMR region by random DNA to restore the plasmid size. But maybe I'm missing something here.

We have reformulated this section to stay closer to our experimental data (l330, “deletion ameliorates plasmid cost in high PCN plasmids”). We do believe such effects arise from the metabolic costs associated to the high gene dosage of the AMR region when in high PCN plasmids, but agree that mutants with varying plasmid size and gene content independently would ultimately be more informative. We have added a line in the discussion (l517) to mention varying plasmid length (in the context of its effect on horizontal transmission).

Line 287 and Fig. 6 - this section is very interesting but I find it also somewhat misleading. The 'stealth plasmid' is a variant of R1, and the results show that all R1 variants are 'blocking' transfer. My conclusions would be that inhabitant plasmids (i.e., surface exclusion mechanism) block plasmid transfer better than defense mechanisms that operate upon invasion (and not as the authors state in the title of that section). This is somewhat expected. It would be good to add here a control with the 'wt' R1 - which should also block the transfer; i.e., this is probably not a unique aspect of the 'stealth' plasmid. We fully agree with that interpretation and did not mean to suggest that the behaviour of the ‘stealth’ plasmids was different from any other R1 variant. We have now re-run experiments including a control with a ‘wt’ R1 variant (R1_TcR_, swapping resistance markers), which confirm that for short-term conjugation assays (Fig. 6A) and one-day kinetics (Fig. 6B). Our revised manuscript now states: “In contrast, s_w_e_ and R1_TcR_ displayed very similar efficiency, suggesting that the deletion of AMR genes itself has little impact on defence efficacy, and defence is overall driven simply by exclusion functions carried by all R1 variants.”

In that same section (Fig. 6) - I realize that the authors are testing for the plasmid presence consistently by the number of AMR CFUs, which was (if I get it right) the source of their 'blind spot' in the publication preceding this current work. Are those the AMR that are in the 'excised' island or other markers of the R1 backbone? If the plasmids they are tracking would experience a similar deletion, are the results still correct?

We have indeed been testing for AMR plasmid presence by plating on antibiotics, and all the AMR genes on the plasmid are within the region which is deleted in the variants – there are no AMR markers part of the backbone. Our evolution experiments suggested that deletion of the AMR region takes more than 8 days to impact population dynamics. In the new plasmid stability experiment (Fig. S4) we also tested for the emergence of the deletion in R1 populations, and did not detect any after 8 days. Thus, at the time scale studied in Fig. 6 (2 days in the previous version, 1 day now), we consider that deletion dynamics can be neglected.

Line 308 - transfer would be more accurate here than 'spread'. Fixed (among other changes to Fig. 6).

Line 336 following: I have trouble understanding how the high PCN has a direct effect on vertical transmission. As stated before, I would be careful to conclude this from their experiments without the respective controls. I do agree, that a higher PCN seems to have an effect on transmission (horizontally and maybe vertically). But I am wondering which reasoning could there be for the vertical transmission advantage. As far as I understand (Brantl, 2007; Lilly & Camps, 2015) the effect of one copA* mutant would not affect the respective plasmid carrying the allele but all plasmid variants present in the cell's plasmid pool.

We fully agree with the reviewer that the precise molecular mechanism by which the high PCN has an effect on vertical transmission is unclear, and needs investigated further in the future. Previous experiments analysing incompatibility between R1 variants and R100 (which shares the same replication mechanism and copA* gene) showed that different high copy mutants of R1 led to different incompatibility dynamics towards R100, with the majority displacing R100 (Nordström et al, 1972 *J Bact*), similar to what we observe, but a minority displaying the opposite behaviour and being preferentially lost despite higher copy number (Uhlin and Nordström, 1975 *J Bact*). Experiments using another related plasmid also confirm that the outcome of competition between variants depends on details of the molecular interactions between secondary structures in the RNA (Brady et al, 1983 *J Bact*). Our Fig. S3B also suggests that the copA* mutants observed here are ones which displace the wt (as they are mostly observed at allele frequencies > 0.5). We have added more discussion and references to the two papers cited above that we had not cited yet, in the discussion (l504-508).

I have no comments on the discussion as the results feel rather 'unstable' to me hence the conclusions may change.

The methods are well described.

References

Zhang, X., Deatherage, D.E., Zheng, H. et al. Evolution of satellite plasmids can prolong the maintenance of newly acquired accessory genes in bacteria. Nat Commun 10, 5809 (2019). <https://doi.org/10.1038/s41467-019-13709-x>

Ahmad, M., Prensky, H., Balestrieri, J., ElNaggar, S., Gomez-Simmonds, A., Uhlemann, A.-C., Traxler, B., Singh, A., & Lopatkin, A. J. (2023). Tradeoff between lag time and growth rate drives the plasmid acquisition cost. Nature Communications, 14(1), 2343. <https://doi.org/10.1038/s41467-023-38022-6>

Brantl, S. (2007). Regulatory mechanisms employed by cis-encoded antisense RNAs. Current Opinion in Microbiology, 10(2), 102-109. <https://doi.org/10.1016/j.mib.2007.03.012>

Dimitriu, T., Matthews, A. C., & Buckling, A. (2021). Increased copy number couples the evolution of plasmid horizontal transmission and plasmid-encoded antibiotic resistance. Proceedings of the National Academy of Sciences, 118(31), e2107818118. <https://doi.org/10.1073/pnas.2107818118>

Lilly, J., & Camps, M. (2015). Mechanisms of Theta Plasmid Replication. Microbiology Spectrum, 3(1), 3.1.02. <https://doi.org/10.1128/microbiolspec.PLAS-0029-2014>

Ripoll-Rozada, J., García-Cazorla, Y., Getino, M., Machón, C., Sanabria-Ríos, D., de la Cruz, F., Cabezón, E., & Arechaga, I. (2016). Type IV traffic ATPase TrwD as molecular target to inhibit bacterial conjugation. Molecular Microbiology, 100(5), 912-921. <https://doi.org/10.1111/mmi.13359>
