# Supplementary material for: Plasmid streamlining drives the extinction of antibiotic resistance plasmids under selection for horizontal transmission
Source: PLoS Biol. 2025 Dec 11;23(12):e3003564. doi: 10.1371/journal.pbio.3003564 (PMC12711027; doi:10.1371/journal.pbio.3003564)
Supplement: Attachment — Submitted filename: response_to_reviewers.docx [file pbio.3003564.s017.docx]

Reviewer #1: Overall, I am mostly satisfied with the revisions the authors have made to the manuscript. I am happy to see a model that helps to generalise and extend the main findings, and highlights some interesting areas for follow-up studies.

We thank the reviewer for their positive comments and suggestions.

There remain some (relatively minor) outstanding issues, as I indicate below.

- Line 119. Clarify. In the text above "isolated one plasmid clone" but here referring to "populations" again. May need a few words just to clarify, e.g. "heterogeneity in the population emerging from the single clone" Added
- Line 120ff. I wonder if this section could be clarified further. "We took advantage of control populations from our evolution experiment, which had been established without any plasmids, and for which we had sequenced clones without any antibiotic selection step". "Plasmid-free" is not correct, as you describe a plasmid in this population. Fixed – replaced ‘plasmid-free controls’ by ‘control populations established without plasmid’’

- Line 136ff dissects the PCR methodology to identify streamlined plasmids. In my opinion, and in the context of this paper, extended dissection of these PCR artefacts seems to be a digression into technical curiosities (i.e. template switching) rather than relevant results, and might be better placed in the methods or supplemental information. We agree and have moved the discussion of PCR artefacts to the methods section.
- I agree with the authors that there are visible differences in band intensity, and even though the 'faint band' is a bit too intense for my liking (particularly in S2D top row), the controls included in these experiments means that I trust the results shown e.g. in Fig. 2. Thank you. The PCR conditions for Fig. S2D were chosen specifically to make sure the results for the ‘longer’ primer pair (with longer product) are as robust as possible, so we used 35 cycles of amplification, which leads to a much more intense band for the R1_del_ primers – but these conditions were never used for screening colonies from evolution/competition assays, which all used only 30 cycles.

The only issue comes when there are heterotypic cells containing both plasmid variants, there might be a spectrum of intensities making it difficult to distinguish low-frequency deletants from the spurious signal from full length plasmids. The authors should ideally validate key results in Fig 2 by repeating a subset with the longer, more stringent primer pairs (though I note a faint band in the second R1 lane in S2D bottom row...). We agree with the reviewer that signal from heterotypic cells would be unclear. However, we believe this should minimally affect results from Fig 2 because at the end of the experiment, AMR plasmids were present at low density (if at all) in most populations, thus the large majority of colonies tested will contain either no plasmid or only streamlined variants. Heterotypic cells do not persist for long as the high-copy number variants rapidly displace the low-copy number ones (see Fig. 4), and Illumina sequencing data suggests the variants are already displacing the ancestral plasmid in midpoint clones (which themselves are part of the small proportion of cells, compared to the whole population, which still express AMR).

More generally, Fig. 2 main insight is to show that streamlined plasmids spread through the whole population, and the possibility of missing some low frequency deletants only makes the test more conservative.

I also wonder if the authors could develop an alternative assay whereby the reverse primer is located within the deleted region (thus giving a band for the non-streamlined plasmid) which might be more robust to template switching. A design might be implemented whereby three
primers are used, with a common 'outside' primer, an AMR-internal-region primer that generates a smaller product for the wild-type plasmid, and a primer at the other end of the deleted region that gives a larger product for the streamlined plasmid. This design would likewise suffer from low sensitivity at detecting the deletants. However, one advantage would be that for clones dominated by full-length plasmids, competition between amplicons (favouring shorter amplicons) would mean that full-length plasmid clones would generate primarily/only the shorter amplicons, titrating out the low-frequency artefacts, and giving a strong positive signal for the continued presence of the full-length plasmid in the tested population. This may be excessive for the current manuscript but could be considered in future studies. We thank the reviewer for the suggestion. We have been considering various PCR designs for the future but are favouring an alternative approach based on adding back a resistance or fluorescence marker to the streamlined plasmids, allowing us to study co-existence without the artefacts we see with PCR.
- Line 139. The authors might like to indicate here that the alternative hypothesis — that the variant emerged by transposase-mediated deletion between the outer repeats of the IS1 copies — is not supported by the retention of a copy of IS1. We have added a mention of that alternative hypothesis, contrasting it to homologous recombination.
- The mathematical model is a nice addition to the paper and highlights the importance of plasmid-plasmid competition in the spread of traits. The authors might like to make it clearer that the role of streamlined plasmids in helping cells resist AMR plasmids is premised on the infectious transmission of AMR, rather than under selective conditions. For example, in the abstract line 86. Next, it would be interesting to look at how streamlined plasmids affect AMR invasion under conditions where selection for full-length plasmids is applied, or applied in a fluctuating or spatial-structured manner… (I'm sure the authors have this idea in mind already!) Adding antibiotics back into the evolution experiment is indeed one of the next things to test! We have added in the discussion a mention of the fact that our study focuses only on non-selective conditions / the absence of antibiotics, and that selection for full-length plasmids would likely favor AMR plasmids by direct selection and by limiting infections transmission.

- Line 232 — suggest reword: "are expected to be primarily driven by competition rather than evolutionary dynamics." Evolutionary dynamics can still play an important role in the short term. Changed, thank you.
- It is perhaps a bit unconventional for gamma to indicate death in the model, rather than conjugation, but it's clearly signposted, so I don't mind too much.

- Important to define 'vertical transmission'. Vertical transmission often taken to include plasmid cost, but the distinction is made here such that 'vertical transmission' refers mainly to persisting in the face of (or outcompeting) co-resident plasmids, i.e. segregational effects (line 472).  We have clarified that our vertical transmission discussion applies to competition with co-resident plasmids; and also added “in the absence of horizontal transmission” to our discussion of previous studies looking at both within- and between-host competition. We agree that more generally, there would be a case for clarifying the interplay between the three layers of within-cell vertical, between-cell vertical (cost) and horizontal transmission, but do not want to discuss this too much here as we have relatively little data or focus on the cost component.

Reviewer #2: The manuscript 'Plasmid streamlining drives the extinction of antibiotic resistance plasmids under selection for horizontal transmission' by Matthews, Lehtinen & Dimitriu is now much clearer compared to the previous version. I commend the authors for their efforts in improving the clarity and presentation.
The authors have addressed all of my previous questions and have revised the manuscript accordingly. In my view, they have also responded thoroughly to the major suggestions and criticisms raised by the other two reviewers.

We thank the reviewer for their positive comments.
I have only a few minor comments:
(Lines of the new version - the one without track-changes)
Introduction, Lines 68-70, I'd suggest joining these two sentences. Something like: "We previously studied experimentally the short-term evolution of R1, a model conjugative plasmid conferring resistance to multiple antibiotics, and one of the first and best studied conjugative plasmids (16,17). " Changed, thank you.
Results section, line 110: I could not understand this: "dropped to values either higher than the ancestral plasmid, …". Thank you, we have reformulated this, as the “drop” was in comparison to the rest of the sequence, making it confusing. It now reads “Depending on the clones, relative coverage in this region was either higher than the one of the ancestral plasmid, equal to the ancestral plasmid (2-3 copies per chromosome), or lower than the ancestral plasmid or even than the chromosome”.
Legend of Fig. 1: I'd suggest including some explanations here: the meaning of m, w, and the treatments Done.
Several places (e.g. line 477): β-lactamases instead of B-lactamases Fixed.
Methods: how much higher is the mutation rate of mutL mutants? 50x? We have not measured this ourselves but added an estimate from the literature together with a reference.
Line 573: " Iin the experiments shown in Fig. 6…" should be: "In the experiments shown in Fig. 6…" Fixed, thank you.
